# P53 IHC Result as a Prognostic Tool in MDS

**DOI:** 10.30699/IJP.2023.1971023.2991

**Published:** 2023-07-16

**Authors:** Alireza Rezvani, Ahmad Monabati, Zahra Kargar, Akbar Safaei, Mahdi Mahmoodzadeh, Hamideh Moosapour, Marzieh Hosseini, Abdolmajid Taheri, Soleiman Kheiri, Elham Taheri

**Affiliations:** 1 *Department of Hematology, Medical Oncology and Stem Cell Transplantation, Hematology Research Center, Shiraz University of Medical Sciences, Shiraz, Iran *; 2 *Department of Pathology, Molecular Pathology and Cytogenetic Ward, Shiraz University of Medical Sciences, Shiraz, Iran *; 3 *Molecular Pathology and Cytogenetic Ward, Pathology Department, School of Medicine, Shiraz University of Medical Sciences, Shiraz, Iran*; 4 *Department of Molecular Pathology & Cytogenetics, Shiraz University of Medical Sciences, Shiraz, Iran *; 5 *Department of Hematology, Isfahan University of Medical Sciences, Isfahan, Iran *; 6 *Evidence-Based Medicine Research Center, Endocrinology and Metabolism Clinical Sciences Institute, Tehran University of Medical Sciences, Tehran, Iran *; 7 *Molecular Pathology and Cytogenetic Ward, Department of Pathology, School of Medicine, Shiraz University of Medical Sciences, Shiraz, Iran *; 8 *Department of Radiology, School of Medicine, Hajar Hospital, Shahrekord University of Medical Sciences, Shahrekord, Iran *; 9 *Department of Epidemiology and Biostatistics, School of Health, Modeling in Health Research Center, Shahrekord University of Medical Sciences, Shahrekord, Iran*

**Keywords:** IHC, Myelodysplastic syndrome, Prognosis, Tumor Suppressor Protein p53

## Abstract

**Background & Objective:**

Some of the patients with myelodysplastic syndrome (MDS) are categorized as good prognosis based on the Revised International Prognostic Scoring System (IPSS-R). However, these patients may have poor clinical outcomes. It seems that the current diagnostic tools and IPSS-R cannot consider genetic factors for determining the prognosis of MDS patients.

**Methods:**

This cross-sectional study included all adult MDS patients of both genders who were admitted from March 2015 to March 2020 to the Hematology wards of two educational tertiary hospitals in Iran (Namazi and Faghihi, affiliated with Shiraz University of medical sciences). Study data included relevant retrospective data from medical records and the results of immunohistochemical p53 staining on bone marrow biopsies.

**Results:**

Of the 84 patients, 65 (77.4%) showed p53 expression in bone marrow. They had shorter median survival than those without p53 expression. Considering both variables of P53 IHC results and IPSS-R score, the patients who died with low-risk IPSS-R score presented high p53 expression.

**Conclusion:**

This study shows that the investigation of p53 expression by IHC at the time of diagnosis is a valuable indicator of survival rate in MDS patients. These data suggest that the immunohistochemical analysis of p53 can be a prognostic tool for MDS and should be used as an adjunct test to make decisions on the best therapeutic choice.

## Introduction

Myelodysplastic syndromes (MDS) constitute a diverse category of diseases marked by the inability of hematopoietic progenitor cells to differentiate properly (1). MDS patients have an increased risk of developing secondary acute myeloid leukemia (AML), characterized by dysplasia in one, or more myeloid cell lineages and consequently cytopenia in peripheral blood cells (2). Older age, chemotherapy, radiation, and environmental factors such as smoking are risk factors for MDS (3, 4). Cytogenetic abnormalities such as monosomy 7, trisomy 8, and deletion of the long arm of chromosome 5 (del5q) have also been found in 50% of cases with MDS (5). Familial studies suggest that genetic factors play a role in the development of MDS (6).In the last decade, various sequencing methods have made a decisive contribution to gaining important insights into the etiology and genetics of MDS (7, 8).

Molecular studies have consistently shed light on cancer-related processes such as DNA repair and apoptosis in MDS development (2). Several studies have suggested p53 mutations as an independent prognostic factor in human MDS (3, 4). Mutations in the TP53 gene, a crucial component in DNA repair and apoptosis, are a constant element of phenotypic diversity in MDS mouse models (12). The p53 mutation is most common in patients with high-risk MDS, although it is also found in patients with isolated del5q and patients with complicated karyotypes (5). Although there appears to be a correlation between the expression of p53 and overall survival (OS) (14). Several clinical and molecular variables have a predictive value and may be more appropriate for accurate stratification of MDS patients.

The International Prognostic Scoring System (IPSS) is a method of risk stratification used to assess overall survival and time to onset of AML in MDS patients. As a standard clinical tool, IPSS stratify patients based on the peripheral blood cytopenia, the patient karyotype at diagnosis, and the percentage of bone marrow blasts (15). This information aid in categorizing the patient into one of four groups: low, intermediate, intermediate, and high risk (6). The IPSS has some drawbacks, including the relative overrepresentation of the blast fraction, underestimation of risk in some patients with severe cytopenia or a normal karyotype, and under-representation of cytogenetic abnormalities.

The revised IPSS (IPSS-R), assessing OS and the rate of AML transformation, integrates more cytogenetic data than the original IPSS and better stratifies relevant prognostic risks(16). Based on the IPSS-R, prognostic groups can be distinguished as the median OS ranges between 0.7 years (very high risk) to 5.4 years (extremely low risk) (17). The treatment options are typically adopted based on risk stratifications such as IPSS-R (7). The treatment goals for low-risk patients are to reduce transfusion dependence and improve quality of life; however, the goal for high-risk patients is to improve overall survival (OS) and slow AML progression (19).

This study aimed to evaluate the prognostic value of p53 protein expression and its relations with IPSS-R score, OS, chromosomal abnormalities, and clinical factors, by the use of IHC.

## Material and Methods

**Patients**

In this cross-sectional study, all adult MDS patients of both genders were admitted from March 2015 to March 2020 to the Hematology wards of two educational tertiary hospitals in Iran (Namazi and Faghihi, affiliated with Shiraz University of medical sciences). Diagnosis of MDS was made according to World Health Organization (WHO) criteria. As a consensus, the minimum diagnostic criteria were as follows: marked and persistent peripheral cytopenia (>6 months) in at least one major hematopoietic lineage, MDS-associated bone marrow features (i.e., one or more of the following features in at least one major hematopoietic lineage: dysplasia ≥ 10%, ring sideroblasts (RS) ≥ 15%, or myeloblasts ≥ 5%, or an MDS-associated karyotype. Moreover, all other hematopoietic and non-hematopoietic disorders were ruled out as the primary causes of dysplasia and/or cytopenia.

**Data Collection**

Clinical data were derived from the patient's medical records including age, gender, contact number, date of diagnosis, as well as cytogenetic study, complete blood count (CBC) (hemoglobin, white blood cells (WBC), and platelet), and bone marrow biopsy (blast, differential count, fibrosis, RS, iron store) records at the time of diagnosis.

**IHC Analysis**

Immunohistochemical analysis of p53 was carried out according to the standard protocol. Bone marrow biopsy sections were available in the form of paraffin-embedded blocks. They were deparaffinized, hydrated, and washed with buffered saline (pH=7.0). Subsequently, antigen retrieval was performed using 10 mm citrate buffer (pH=6.0) for 30 minutes, and then the sections were treated twice for 5 minutes each time with a methanol solution containing an endogenous peroxidase and 0.03% hydrogen peroxide. The sections were then incubated with a p53-specific monoclonal antibody (Clone DO-7) for 12 hours at 4°C. The slides were then washed with buffered saline and incubated for one hour with biotinylated IgG antibody after which the sections were again washed with buffered saline and incubated with the ABC complex (DAKO) for 45 minutes. The ABC complex contains 5 μL avidin and 5 μL biotin in 5 mL buffered saline. To visualize the reaction, the slides were treated with a 1 mg/mL diaminobenzidine solution, followed by counterstaining with hematoxylin. Coverslips were attached to the slides using Canada Balsam. The p53 protein expression was defined as being positive or negative based on the level of nuclear staining. A positive value was indicated if at least 1% of the hematopoietic cells showed nuclear staining.

**Ethical Considerations**

Patients who were admitted to the two major university hospitals and implied their consent to the use of their clinical records retrospectively for research purposes entered the study. However, the principal researcher called the patients to follow them up and complete the data; in the first contact, she explained the subject and goal of the study to them. The patients enrolled gave oral and written consent to participate in the study. They were assured that their privacy and data confidentiality would be protected, and they could be withdrawn from the study at any time they want.

In addition to the retrospective clinical data, available in hospital records, p53 IHC studies were required for research purposes. These tests were done using the patient's bone marrow samples, routinely taken for diagnosis, and then stored in paraffin blocks. Patients, therefore, did not need to undergo repeated bone marrow biopsies or suffer additional risks or harm for research purposes. Additionally, strategies such as coding were used for data registration and analysis to protect personal privacy and confidentiality.

**Data Analysis**

± and interquartile range (IQR) for non-normally distributed quantitative variables. Normal distribution was tested using Shapiro–Wilk test. Group comparisons were analyzed using the tests of independent T, Mann–Whitney U, Chi-square, or Fisher's exact as appropriate. Survival times were compared using the tests of Kaplan-Meier and Log-rank. A Cox proportional hazards regression model was applied to examine the association between P53 IHC results and IPSS-R scores in the absence of death. Statistical significance was defined as a P-value< 0.05 for all tests.

## Results

Of 120 patients with a condition called MDS admitted to the hematology wards of Namazi and Faghihi hospitals, 84 patients fulfilled the inclusion and exclusion criteria. The paraffin blocks didn’t have sufficient bone marrow biopsy material for 36 patients, so they were excluded from the study. Contact numbers for three patients were not available. All of these patients gave consent to participate in the study. Thirteen patients did not answer the researcher’s telephone calls; consequently, parts of the data (including the survival status, smoking, the need for blood perfusion, and the job) were not available for these patients. 

Based on the current WHO classification, the patients were distributed in distinct subtypes of MDS as follows: the frequencies were 6, 8.3, 23.8, 3.6, 27.4, 23.8, 4.8, and 2.4% for subtypes of Del5q, Excess blast-1, Excess blat-2, MDS-U, MDS with multilineage dysplasia, MDS with single lineage dysplasia, RS-MLD, and RS-SLD, respectively. Sixty-five patients (77.4%) showed p53 IHC-positive results Table 1 shows the demographic and para-clinical characteristics of 84 patients enrolled in the study based on the IHC studies. 

**Table 1:** Demographic and Clinical Characteristics of the patients with diagnosis of MDS admitted in Hematology wards

		P53 expression		P-value
		Negative (n=19)	Positive (n=65)	
Age (Mean ± SD)		54.11 ± 3.73	59.62 ± 1.93	0.18
Gender	Male	14 (25.5%)	41 (74.5%)	0.39
	Female	5 (17.2%)	24 (82.8%)	
Cytogenetic analysis	Normal	10 (11.9%)	9 (10.7%)	0.98
	Chromosome abnormalities	34 (40.5%)	31 (36.9%)	
IPSS-R risk N (%)	Very low	2 (2.4%)	2(2.4%)	0.02^*^
	Low	6 (7.1%)	13(15.5%)	
	Intermediate	9 (10.7%)	18(21.4%)	
	High	2 (2.4%)	12(14.3%)	
	Very high	0	20(23.8%)	
CBC #	Hemoglobin	8.8 (6.6-10.7)	7.9 (6.8-9)	0.3
	WBC	3300 (2300-5600)	3200 (2150-5350)	0.78
	Platelet	100000 (54000-185000)	64000 (26500-121000)	0.05
	Absolute Neutrophilic count	1.38 (0.54-2.6)	1.5 (0.61-2.5)	0.85
BoneMarrow Aspiration	Blast	2 (2-4)	4 (2-11)	0.04

^ Namazi and Faghihi hospitals

# Median (IQR)

**Analysis of the Relation Between p53 IHC and Other Factors**

Comparing P53 IHC-positivity among patients with high iron storage in bone marrow showed that the majority of them were IHC-positive for p53; however, this was not statistically significant (76. 6% vs 23.8%, *P*= 0.62).

In P53 IHC-positive patients compared to negative patients, there was a significant frequency of need to blood perfusion (80.7%vs 19.3%, *P*=0.008).

Regarding the frequency of active or passive smoking, it was higher in P53 IHC-positive patients than those in P53 IHC-negative patients; however, the difference was not statistically significant (42.3 vs 26.3%, *P*=0.22).

**Analysis of the Survival Rate**

Considering P53 IHC results, the survival rate in P53 IHC-positive patients was lower than those in negative patients (5.7% vs. 94.7%, *P*<0.001).

Considering the IPSS-R score, the survival rate of the patients in different groups of IPSS-R score is listed in Table 2. The death was significantly related to the IPSS-R score (Fisher’s exact test, *P*=0.016). Table 2 shows that the overall survival rate was low even in IPSS-R low-risk patients (33.3%).

Considering both variables of P53 IHC results and IPSS-R score, dead patients with low-risk IPSS-R scores showed more positive p53 results (Table 2).

**Table 2:** Analysis of the survival rate based on the IPSS-R, both variables of IPSS-R and P53 IHC, and cytogenetic results.

		Dead	Alive	
		N (%)	N (%)	P-value
IHC P53 results				
Positive IHC		50(94.3%)	3(5.7%)	<0.001
Negative IHC		1(5.7%)	18(94.7%)	
IPSS-R Score				
Very low risk		2(50%)	2(50%)	0.016
Low risk		12(66.7%)	6(33.3%)	
Intermediate risk		14(58.3%)	10(41.7%)	
High risk		7(70%)	3(30%)	
Very high risk		16(100%)	0	
Cytogenetic results				
Very poor		8(11.1%)	0	0.57
poor		1(1.4%)	1(1.4%)	
Intermediate		14(19.4%)	6(8.3%)	
good		28(38.9%)	12(16.7%)	
Very good		0	2(2.8%)	
IPSS-R Score and IHC P53 results				
Very low risk	Positive IHC	2(50%)	0	0.08
	Negative IHC	0	2(50%)	
Low risk	Positive IHC	12(66.7%)	0	
	Negative IHC	0	6(33.3%)	<0.001
Intermediate risk	Positive IHC	13(54.2%)	2(8.3%)	
	Negative IHC	1(4.2%)	8(33.3%)	<0.001
High risk	Positive IHC	7(70%)	1(10%)	
	Negative IHC	0	2(20%)	0.06
Very high risk	Positive IHC	16(100%)	0	
	Negative IHC	-	-	-

**Analysis of the Survival Time**

**Fig. 1:**
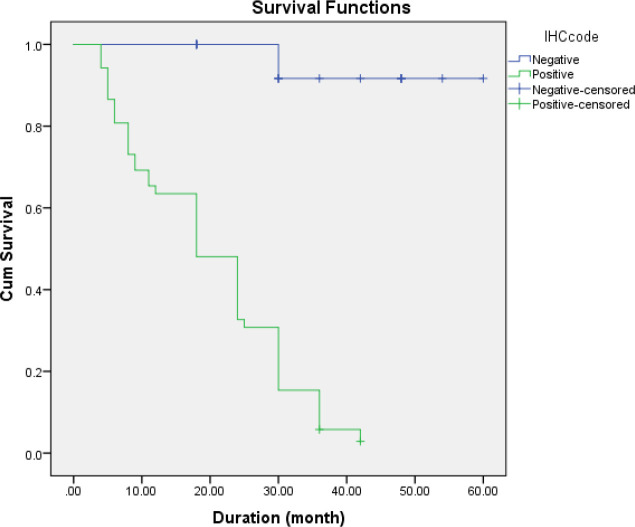
The Kaplan-Meier survival curve for P53 IHC-positive and negative patients

Based on the karyotype results, patients’ survival rate was also compared. The Kaplan-Meier survival curves for both patient groups are shown in Figure 1. Considering P53 IHC results, the mean survival time of the positive patients was significantly lower than that of negative patients (20 (95% CI; 16.8-23.2) vs. 57.5 (95% CI; 52.8- 62.19) months, *P*<0.001).

Based on the Cox proportional hazards regression model, of the P53 IHC results and IPSS-R score variables, only the P53 IHC results were significantly associated with mortality (hazard ratio (HR), 268; 95% CI, 25-2898). In other words, despite information on the P53 IHC results, the IPSS-R score variable does not include more information on mortality.

## Discussion

Despite the IPSS-R scoring system, it is difficult to assess all the factors that influence a patient's prognosis (20). In addition, commonly used prognostic scoring systems do not include the examination of certain molecular genetic features, especially single-gene mutations. Previous studies have revealed that p53 expression can predict the development of acute leukemia and OS in MDS patients (21-23).

In this study, the mean OS time in the p53-positive and p53-negative groups was 57 and 20 months, respectively. Our data identified 68.4, 66.7, 85.7, and 100 percent of the patients with p53 expression in the low, intermediate, high, and very high-risk groups, respectively. Our rate was higher than that in the cohorts of two different groups independently studied by Saft *et al.* (24), and Oliva *et al.* (25)**. **Duarte *et al.* studied 38 patients with low-risk MDS and found that those with positive p53 expression had a poor prognosis (23). Similarly, increased p53 expression in 85 MDS cases with del5q was associated with a higher likelihood of AML formation, shorter OS, and lower cytogenetic response rate to lenalidomide treatment (24). Another study revealed that OS was significantly worse when more than 50% of cells showed p53 expression (26). Furthermore, in a group of 67 MDS patients, high levels of p53 expression (≥10% of the cells) were associated with TP53 mutations, increased BM blast counts, low-risk karyotypes, and most importantly, decreased OS (27).

The true frequency of TP53 mutations is underestimated, but IHC overexpression of p53 is always a marker for a molecular alteration with a poor prognosis (28). Since TP53 mutations cause protein stabilization and accumulation, the detection of nuclear p53 by IHC has been used as an alternative diagnosis for TP53 mutations in hematologic malignancies (29,30). Nuclear expression of p53 was also associated with TP53 mutations and prognosis in patients with relapsed myeloma treated with lenalidomide (31). Similarly, overexpression of p53 in wild-type TP53 is considered a simple diagnostic method to detect a TP53 mutation. McGraw *et al.* reported 100% specificity and 60% sensitivity of IHC for p53 overexpression in detecting TP53 mutations (32). According to a study by Jaedersten *et al.*, the presence of more than 2% BM progenitor cells with high p53 staining is associated with TP53 mutation (33). However, IHC has the disadvantage of not detecting nonsense mutations that cause a truncated and unstable protein and occur in 10% of high-risk MDS patients (34).

Several biochemical parameters such as ferritin, albumin, hemoglobin, ApoA1, 2-microglobulin, and platelet count, as well as age, have a significant impact on patient health status and survival (35-39). Even though the IPSS-R is the gold standard for the management of MDS patients, it doesn’t assess all of the biochemical characteristics that contribute to patient prognosis prediction. We found a correlation between the expression of p53 and a lower number of platelets and a higher number of ring sideroblasts and blasts in BM of MDS patients. Due to functional platelet abnormalities caused by RUNX1 deficiency, familial platelet disease is manifested by severe bleeding and an increased risk of developing AML and MDS(40). Defective platelet aggregation was also highly associated with poor outcomes in a study of 26 MDS patients (41).

Although there was no statistically significant difference in chromosomal abnormalities between the p53-positive and p53-negative groups, previous studies have shown that multiple chromosomal abnormalities are associated with a more rapid progression of MDS and leukemic transformation (42). Moreover, cytogenetic abnormalities are more common in high-risk MDS patients, and more abnormalities are observed in aggressive MDS than in benign MDS (43,44). Similarly, in 60 MDS patients treated with Aza, Nishiwakia *et al.* examined the prevalence of p53 expression as a predictive factor. They discovered that lower OS along with poor cytogenetics was significantly more common in p53-positive patients than in p53-negative patients (45).

Overall, we demonstrated the prognostic function of p53 protein expression in MDS patients with very high, high, intermediate, and low-risk IPSS-R. IHC detection of p53 was able to provide a short-term prognosis independent of the IPSS-R score. Compared to the molecular detection of TP53 mutations by PCR or NGS techniques, p53IHC is more cost-effective. Compared with previous studies, our study included more participants in all IPSS-R risk groups; however, these findings need to be reproducible in more studies to support the above evidence.

## Conclusion

This study shows that the evaluation of p53 expression by IHC at the time of diagnosis is a useful indicator of survival rate in MDS patients. Despite the risk of IPSS-R, we suggest that p53IHC should be routinely used as a prognostic test in MDS patients. As a less expensive and more accessible method, it has the potential to help physicians to make more precise and individualized decisions for MDS patients in our country where healthcare resources are inadequate.

## Funding

None.

## Conflict of Interest

The authors declared no conflict of interest.
